# Achieving good adherence to inhaled corticosteroids after weighing canisters of asthmatic children

**DOI:** 10.12688/f1000research.10710.1

**Published:** 2017-03-14

**Authors:** Wantida Chuenjit, Vorapan Engchuan, Araya Yuenyongviwat, Pasuree Sangsupawanich

**Affiliations:** 1 Division of Allergy and Immunology, Department of Pediatrics, Faculty of Medicine, Prince of Songkla University, Hat Yai, Songkhla, Thailand

**Keywords:** canister weight, adherence, compliance, inhaled corticosteroids, asthma, wheezing, children, cohort study

## Abstract

**Background: **The metered-dose inhalers (MDIs) currently available for inhaled corticosteroid delivery do not offer an integrated dose counter; therefore, it is difficult to evaluate adherence of patients. The present authors developed a linear regression equation using canister weight to calculate the number of doses actuated from the MDIs. This study aimed to assess medical adherence after the integration of regular weighing of the canisters into the routine service. **Methods:** A cohort study was carried out between May 2013 and April 2014. Children aged less than 8 years with a diagnosis of asthma were recruited. The duration of adherence assessment was 24 weeks. Participants had a regular schedule every 8 weeks to obtain a new FLIXOTIDE® 125 inhaler. Parents were asked to collect the discarded MDI canisters, which were then weighed by a laboratory scale. The weight of each canister was replaced in the regression equation to calculate the number of doses actuated from the MDIs. **Results:** A total of 52 asthmatic children participated in the study. The median age was 52.7 months. At the end of 24 weeks, 44, 33, and 23 discarded MDI canisters were collected from visits 1, 2, and 3, respectively. The median percentages of adherence were 96.8%, 96.3%, and 96.3%, respectively. In 11 discarded canisters (11%), the remaining medication was more than 30% of the labeled doses. Approximately 90% of the participants had no asthma exacerbation during 24-week study period. **Conclusion:** High adherence rates were achieved after integration of canister weighing into the asthma care service.

## Introduction

Inhaled corticosteroids (ICSs) are the standard treatment for asthmatic children. Non-adherence with prescribed ICS treatment clearly causes uncontrolled asthma ^1^. Feedback from parents is the traditional approach to assess the adherence to the treatment regimen; however, there is an overestimation by the patients of the remaining amount of medication ^2^. Even though integration of a dose counter into the inhaler device improves the tracking adherence to prescribed medication ^3^, the metered-dose inhalers (MDIs) currently available for ICS delivery do not offer integrated dose counters.

Weighing of the MDI canisters may be an alternative method to assess a patient's medication adherence. The present authors previously developed a linear regression equation using canister weight to calculate the number of doses actuated from the MDIs ^4^. Weighing of the canisters was implemented into our asthma care system in March 2013. This study was designed to assess a patient's medication adherence after the integration of regular weighing of the canisters into the routine service.

## Methods

### Study design and subjects

A cohort study was carried out between May 2013 and April 2014. The inclusion criteria were children aged less than 8 years with a diagnosis of asthma who attended the Pediatric Allergy Clinic at Songklanagarind Hospital (Hat Yai, Songkhla, Thailand) and had exacerbation of asthma requiring hospitalization or an emergency department visit within the previous year. Patients who had a previous history of intubation or other chronic conditions were excluded.

The research protocol (REC 55-021-01-1-2) was approved by the Human Research Ethics Committee, Faculty of Medicine, Prince of Songkla University. Informed consent was obtained from the parents/guardians.

### Asthma management

Fluticasone propionate was selected as the ICS therapy for the study. A FLIXOTIDE® 125 Inhaler (GlaxoSmithKline) is a pressurized metered-dose inhaler, which delivers 125 microgram of fluticasone propionate per actuation. Each canister supplies 120 actuations. FLIXOTIDE® 125 Inhaler and BabyHALER® (GlaxoSmithKline), a device to help patients taking inhaled medicine, were prescribed to all participants. The dosage of fluticasone propionate was one actuation twice a day. The add-on asthma therapies were provided according to the GINA guideline ^5^. Participants needed to participate in a regular schedule every 8 weeks to obtain a new FLIXOTIDE® 125 inhaler. For patients who did not achieve adequate control or maintain the adherence rate, the inhalation technique and medication doses were revised at the time they visited the clinic. Exacerbation was defined as asthma deterioration that required treatment with systemic corticosteroids or emergency department utilization or hospitalization.

### Adherence assessment

The duration of adherence assessment was 24 weeks. Each participant received three canisters of FLIXOTIDE® 125 Inhaler. Parents were asked to collect the discarded inhalers at the 8-week (visit 1), 16-week (visit 2), and 24-week (visit 3) after recruitment. The discarded MDI canisters were weighed by a laboratory scale (Sartorius Basic®). The weight of each canister was replaced in the regression equation to calculate the number of doses actuated from the MDIs. A regression equation for a fluticasone propionate MDI canister gives the number (n) of doses actuated from the MDIs:

              n = 276.16 – (14.62 × canister weight). ^4^

### Statistical analysis

All of the statistical analyses were conducted with R software (version 3.3.2) by the R Foundation for Statistical Computing. Adherence in each 8-week interval was calculated as the amount of medication actuated divided by the amount prescribed. Percentage of adherence was reported as median and range.

## Results

A total of 52 asthmatic children participated in the study. The characteristics of the participants are shown in Table 1. Half of the participants were male. The median age was 52.7 months (range, 18.3–91.7) and the age at the onset of asthma was 12 months (range, 1.0–48.0). Parents were the major caregivers. In total, 32% of the participants had other allergic co-morbidities. Most of participants had received ICS therapy for longer than 3 months. At the end of 24 weeks, 44, 33, and 23 (total 100) discarded MDI canisters were collected from visits 1, 2, and 3, respectively. The remaining median weights of the discarded canisters from visits 1, 2, and 3 were 11.172g, 11.229g, and 11.113 g, respectively, and the median percentages of adherence were 96.8%, 96.3%, and 96.3%, respectively. In 11 discarded canisters (11%), the remaining medication was more than 30% of the labeled doses. Approximately 90% of the participants had no asthma exacerbation during 24-week study period ( Table 2).

**Table 1.** Demographic data of asthmatic children.

Variable	Results (N=52)
Male, n (%)	29 (55.8)
Weight, kg, median (range)	18.2 (10.9–30.0)
Height, cm, median (range)	107.4 (84.6–127.5)
Age, months, median (range)	52.7 (18.3–91.7)
Age onset, months, median (range)	12 (1.0–48.0)
Allergic disease, n (%) • Atopic dermatitis • Allergic rhinitis • Food allergy	17 (32.7) 3 (5.8) 12 (23.1) 5 (9.6)
Caregiver, n (%) • Parents • Grandparents	44 (84.6) 7 (13.5)
Number of hospitalizations, times/ person, median (range)	1 (1–5)
Number of exacerbations within previous year, times/person, median (range)	3 (1–9)
Duration from last exacerbation, months, median (range)	4 (3–11)
Duration of ICS therapy, n (%) • 0–3 months • More than 3 months	15 (28.9) 37 (71.1)
Number of studied canisters, n (%) • Visit 1 • Visit 2 • Visit 3	44 (84.6) 33 (63.4) 23 (44.2)

**Table 2.** Canister weight and percentage of adherence.

Variables	Visit 1	Visit 2	Visit 3
Number of canisters	44	33	23
New canister weight, g, median (range)	18.838 (18.663–18.929)	18.819 (18.607–18.907)	18.812 (18.470–18.913)
Discard canister weight, g, median (range)	11.172 (7.889–16.042)	11.229 (7.825–17.146)	11.113 (9.400–16.020)
Actuated doses calculated from canister weight, median (range)	112.8 (41.6–160.8)	112.0 (25.5–161.8)	113.7 (42.0–138.7)
Percentage of adherence, median (range)	96.8% (33.0–143.6)	96.3% (20.2–126.4)	96.3% (36.8–118.7)
Number of participants with exacerbation	1	2	1

Canister weight and percent of adherence Raw data of canister weight (original and discarded weight), results of the actuated dose equation and percent of adherence. Click here for additional data file. Copyright: © 2017 Chuenjit W et al. 2017 Data associated with the article are available under the terms of the Creative Commons Zero "No rights reserved" data waiver (CC0 1.0 Public domain dedication).

## Discussion

The present study demonstrated high adherence rates with low variations between the three visits. The percentage of discarded canisters, which had more than 30% of the labeled dosage of medication remaining, reduced from 22% in our previous cross-sectional study ^6^ to 11% in this study. Achieving good adherence in this cohort could be explained by the Hawthorn effect: the parents and participants knew that their adherence would be measured by weighing the canisters; therefore, the individuals modified or improved their adherence in response to the awareness of being observed.

Approximately 90% of the participants had no asthma exacerbation throughout the study period. In our previous study, only 59% of the patients had adequate control ^6^. Patients had access to the same educational and medication intervention, but the adherence rates were significantly different. Previous studies verified that the adherence rates had an association between lower adherence rates and poor asthma control ^7, 8^.

Although weighing of canisters was less accurate than a dose counter for measuring adherence ^9^, the present study demonstrated that a weight-remaining dose correlation could be used to determine the inhaler medication adherence in real life, and intensive monitoring of adherence was successful in achieving control.

In conclusion, our results demonstrated that high adherence rates were achieved after integration of canister weighing into the asthma care service. The present study highlighted the need to incorporate a method to monitor medical adherence in clinical practice, which may contribute to adequate asthma control.

## Data availability

The data referenced by this article are under copyright with the following copyright statement: Copyright: © 2017 Chuenjit W et al.

Data associated with the article are available under the terms of the Creative Commons Zero "No rights reserved" data waiver (CC0 1.0 Public domain dedication). 

**Dataset 1: Canister weight and percent of adherence.** Raw data of canister weight (original and discarded weight), results of the actuated dose equation and percent of adherence. doi, 10.5256/f1000research.10710.d150970 ^10^
