# Supplementary material for: Achieving good adherence to inhaled corticosteroids after weighing canisters of asthmatic children
Source: F1000Res. 2017 Mar 14;6:266. [Version 1] doi: 10.12688/f1000research.10710.1 (PMC5553081; doi:10.12688/f1000research.10710.1)
Supplement: Peer review history

# Peer review: Referee response for version 1

**DOI:** 10.5256/f1000research.11547.r24513

**Published:** 2017-08-09

**Authors:** Orathai Piboonpocanun

**Affiliations:** 1 Division of Allergy and Immunology, Department of Pediatrics, Faculty of Medicine, Siriraj Hospital, Mahidol University, Bangkok, Thailand

The authors tried to use canister weight to calculate the number of doses actuated from the MDIs and to assess medical adherence to MDIs.

 There are some issues which need clarification

1. Please clarify why the numbers of collected MDI canisters were not the same in each visit.
2. Please mentioned about patients who had exacerbation. Were they the same patients? Were these patients nonadherence to medication or did they have any specific problem?

I have read this submission. I believe that I have an appropriate level of expertise to confirm that it is of an acceptable scientific standard.

# Peer review: Referee response for version 1

**DOI:** 10.5256/f1000research.11547.r22150

**Published:** 2017-04-24

**Authors:** Bee Wah Lee

**Affiliations:** 1 Department of Paediatrics, Yong Loo Lin School of Medicine, National University of Singapore, Singapore, Singapore

This study evaluated adherence to ICS prophylaxis in asthmatic children by using weight of canister as a means to determine inhaler compliance, by calculating doses used by using a regression equation based on canister weight. The authors conclude that in the absence of an inbuilt dose counter, weighing the canister was a useful means of improving adherence to daily treatment with ICS.

 Detailed comments:

1. There were 52 children participating in the study. However, with each follow up visit (n=3)the number of canisters measure reduced progressively (44, 33, 23). Was there loss to follow up or did these patients fail to return their canisters? Could these patients be used as their comparative group in terms of asthma control over the period of follow up?
2. For the subjects with the 11 canisters that had remaining medication, was their asthma control affected by non compliance?
3. It may be more appropriate to use the term ‘used’ rather than ’discarded’ canisters.
4. Under the paragraph: adherence assessment. Some editing of sentences may improve readability:

Parents were asked to ‘return’ the ‘used inhalers at the 8 week…..

 ‘The weight of the canister was replaced in the regression equation’ to “The number of remaining doses

 of remaining doses was extrapolated by using the weight of the used MDI canisters into the regression equation as shown:……..

I have read this submission. I believe that I have an appropriate level of expertise to confirm that it is of an acceptable scientific standard, however I have significant reservations, as outlined above.

# Peer review: Referee response for version 1

**DOI:** 10.5256/f1000research.11547.r21967

**Published:** 2017-04-21

**Authors:** Wiparat Manuyakorn

**Affiliations:** 1 Division of Allergy, Immunology, and Rheumatology, Department of Pediatrics, Faculty of Medicine Ramathibodi Hospital, Mahidol University, Bangkok, Thailand

The article is well written. However, there is some minor comment.

 1. The authors mentioned that "The metered-dose inhalers (MDIs) currently available for inhaled

 corticosteroid delivery do not offer an integrated dose counter". This statement was true at the time when this study was performed. But currently, there are some MDIs that have dose counter. So this statement needs some amendment.

 2. Method: the authors mentioned that "Participants needed to participate in a regular schedule every 8 weeks to obtain a new FLIXOTIDE® 125 inhaler." And the duration of treatment was 24 weeks. So it means that each participant would receive 3 inhalers. A total of 52 children was participated. So I wonder why only 100 MDI canister was evaluated. It should be 52*3=156?. This may need the clarification.

I have read this submission. I believe that I have an appropriate level of expertise to confirm that it is of an acceptable scientific standard.
